# Outcome for Pediatric Adreno-Cortical Tumors Is Best Predicted by the COG Stage and Five-Item Microscopic Score—Report from the German MET Studies

**DOI:** 10.3390/cancers15010225

**Published:** 2022-12-30

**Authors:** Michaela Kuhlen, Marina Kunstreich, Stefan A. Wudy, Paul-Martin Holterhus, Lienhard Lessel, Dominik T. Schneider, Ines B. Brecht, Denis M. Schewe, Guido Seitz, Christoph Roecken, Christian Vokuhl, Pascal D. Johann, Michael C. Frühwald, Peter Vorwerk, Antje Redlich

**Affiliations:** 1 Pediatrics and Adolescent Medicine, Faculty of Medicine, University of Augsburg, 86156 Augsburg, Germany; 2 Pediatric Hematology/Oncology, Department of Pediatrics, Otto von Guericke University Children’s Hospital, 39106 Magdeburg, Germany; 3 Pediatric Endocrinology & Diabetology, Center of Child and Adolescent Medicine, Justus Liebig University, 35435 Giessen, Germany; 4 Division of Pediatric Endocrinology and Diabetes, Department of Pediatrics, Christian-Albrechts-University Kiel & University Hospital Schleswig-Holstein, 24105 Kiel, Germany; 5 Clinic of Pediatrics, Dortmund Municipal Hospital, 44139 Dortmund, Germany; 6 Pediatric Oncology and Hematology, University Children’s Hospital, 72076 Tuebingen, Germany; 7 Department of Pediatric Surgery and Urology, University Hospital Giessen-Marburg, 35033 Marburg, Germany; 8 Department of Pathology, Christian-Albrechts-University & University Hospital Schleswig-Holstein (UKSH), 24105 Kiel, Germany; 9 Section of Pediatric Pathology, Department of Pathology, University Hospital Bonn, 53121 Bonn, Germany

**Keywords:** adrenocortical carcinoma, adenoma, children, treatment, outcome, risk factors

## Abstract

**Simple Summary:**

Pediatric adrenocortical tumors have a poor prognosis. The EXPeRT consortium recently published recommendations for their management. In this study, we report on 161 pediatric ACT patients registered with the German Malignant Endocrine Tumor studies and explore those recommendations. The median age at the diagnosis was 4.3 years, and the mean follow-up was 4.5 years. The 3-year overall (OS) and event-free survival (EFS) estimates were 65.5% and 50.6%. The clinical presentation and prognosis were defined by age. The OS was impaired for patients aged ≥ 4 years, following the initial biopsy, tumor spillage, and incomplete tumor resection, with unfavorable histology, according to the five-item microscopic score and COG stages III and IV. COG stages III and IV and unfavorable histology were impacted as negative prognostic factors upon the EFS and OS.

**Abstract:**

Background: Adrenocortical tumors (ACTs) encompassing the adrenocortical adenoma (ACA), carcinoma (ACC), and tumors of undetermined malignant potential (ACx) are rare endocrine neoplasms with a poor prognosis. We report on pediatric ACT patients registered with the Malignant Endocrine Tumor studies and explore the EXPeRT recommendations for management. Patients: Data from the ACT patients (<18 years) were analyzed. For the risk prediction, the patients were retrospectively assigned to the COG stages and the five-item score. Results: By December 2021, 161 patients with ACT (ACA *n* = 51, ACx *n* = 19, and ACC *n* = 91) had been reported (the median age at the diagnosis was 4.3 years with a range of 0.1–17.8), with lymph node and distant metastases in 10.7% and 18.9% of the patients with ACC/ACx. The mean follow-up was 4.5 years (with a range of 0–16.7). The three-year overall (OS) and event-free survival (EFS) rates were 65.5% and 50.6%. In the univariate analyses, the OS was impaired for patients aged ≥ 4 years (*p* = 0.001) with the initial biopsy (*p* = 0.016), tumor spillage (*p* = 0.028), incomplete tumor resection (*p* < 0.001), unfavorable histology (*p* = 0.047), and COG stages III/IV (*p* = 0.002). Multivariate analysis revealed COG stages III/IV and an unfavorable five-item score as independent negative prognostic factors for the EFS and OS. Conclusions: Age defines the clinical presentation and prognosis in pediatric ACTs. The outcome is best predicted by the COG stage and five-item score.

## 1. Introduction

Adrenocortical tumors (ACTs) are rare endocrine neoplasms arising from the cortex of the adrenal gland. The estimated incidence is 0.2–0.3 cases per million children and adolescents per year [1,2]. A uniquely high population incidence of 3.4 cases/million children/year is seen in Southern Brazil due to the endemic pathogenic *TP53* p.R337H founder variant [3,4,5,6]. Germline *TP53* variants are reported in 50–65% of children with ACTs and in 95% of those in Southern Brazil [3,7].

Completely resectable tumors have a favorable prognosis if they are small, whereas disease recurs in 50% of patients with large, localized tumors [8,9]. Patients with metastatic disease have a poor outcome, with an overall survival of less than 20% [8,10]. It still remains to be defined which patients benefit from more aggressive therapy in addition to complete tumor resection. Yet, effective therapies for children with advanced and even metastatic diseases are still missing. On the one hand, the overestimation of malignancy is possible since distinguishing highly malignant adrenocortical carcinoma (ACC) from benign adrenocortical adenoma (ACA) in children still is difficult [11].

To identify children with an unfavorable prognosis and, thus, a potential indication for systemic therapy, the use of a pediatric pathology score, i.e., the Wieneke index [12] together with clinical risk factors, was proposed [13,14,15,16]. An initiative by the European Cooperative Study Group for Pediatric Rare Tumors (EXPeRT) defined a subset of ‘high-risk’ tumors, in which the presence of distant metastases and a tumor volume > 200 cm^3^ (without accounting for age and body mass, respectively) were identified as independent factors associated with a poor prognosis [10,17,18]. The American National Cancer Data Base reported age as the most important determinant of disease-specific survival [19]. In this series, young patients were more likely to present with favorable features, including local disease and small tumor size. The International Pediatric Adrenocortical Tumor Registry determined the disease stage, presenting signs of endocrine dysfunction, and age as independent factors associated with a prognosis [6].

To further elucidate the significance of the various clinical risk factors for a prognosis, Zambaiti and colleagues conducted a meta-analysis of clinical prognostic factors in pediatric ACC [13]. They identified an age > 4 years, secreting tumors, an incomplete surgical resection, cut-of-values of the tumor volume (200 cm^3^), tumor weight (100 g or 400 g), and the maximum tumor diameter (<5 cm, 5–10 cm, and >10 cm), a >stage I disease, and the presence of Cushing syndrome as predictors of a poorer outcome. In addition, the metastatic spread is universally considered a highly unfavorable prognostic factor [6,10,19,20].

The role of adjuvant therapy in children with ACCs remains still unclear. In the risk-stratified interventional Children’s Oncology Group’s (COG’s) ARAR0332 study, the failure rates of surgery alone in children with localized large tumors were high (a 5-year event-free survival (EFS) of 53.3%), whereas patients with stage III ACC had an excellent outcome with combined surgery and chemotherapy (EFS 81%) [20]; however, the latter comprised mitotane and resulted in significant toxicity. The outcome for children with metastatic disease remained dismal (an EFS of 7.1%). These data corroborate the results of earlier European groups (TREP, Tumori Rari in Età Pediatrica; FRACTURE, Groupe FRAnCais des TUmeurs Rares de l’Enfant) [2,18]. In 2021, the EXPeRT group within the EU-funded project, PARTNER (the Pediatric Rare Tumors Network—European Registry), published diagnostic and therapeutic recommendations for children and adolescents with ACTs [21]. The EXPeRT recommendations propose using the five-item microscopic score described by Picard and colleagues, especially for the stratification of localized ACTs. The five-item score includes adrenal capsular and venous invasion, tumor necrosis, >15 mitoses, and a Ki67 index of >15%. ACTs with more than two features are considered ‘unfavorable’. 

We report on children and adolescents with adrenocortical tumors (ACTs) registered with the German Pediatric Oncology Hematology-Malignant Endocrine Tumor (GPOH-MET) trial center since 1997. We explored the use of the EXPeRT/PARTNER recommendations for the management of pediatric ACT patients. We aim to contribute to the improvement of individual treatment decisions and discuss the implications of our findings for future clinical strategies, including international, collaborative, prospective, and randomized trials.

## 2. Materials and Methods

Our cohort included all children and adolescents aged 0–<18 years with histologically confirmed ACAs, adrenocortical tumors of uncertain malignant potential (ACx), and ACCs reported to the MET study center between January 1997 and December 2021. Some patients in this case series were reported on previously [22,23].

The GPOH-MET 97 protocol and the GPOH-MET registry were approved by the ethics committees of the University of Luebeck (Approval number 97–125) and Otto-von-Guericke-University Magdeburg (Approval number 174/12), Germany. Informed consent from the patient, parent, or guardian was obtained.

### 2.1. The German MET Studies

Details of the GPOH-MET 97 study protocol, the GPOH-MET registry, and the data collection are provided elsewhere [10,23]. Briefly, no additional therapy was recommended for patients with completely resected stage I, II, and III (T3, N0, M0) tumors (AJCC 7th staging system). Four cycles of chemotherapy (alternating NN-1: vincristine, ifosfamide, doxorubicin, and NN-2: carboplatin and etoposide) with mitotane over a nine-month period were advised for patients with stage III (T1-2, N1, and M0) tumors, while patients with stage IV tumors should have received eight cycles of chemotherapy with mitotane over an 18-month period. The GPOH-MET protocol recommended 2–4 cycles of neoadjuvant chemotherapy with mitotane for patients with primary unresectable tumors. Re-evaluation of operability was advised after each cycle of chemotherapy.

For the purpose of this analysis, we defined tumors as secreting if plasma hormone levels and/or urinary-free cortisol were elevated according to local reference ranges and/or if clinical signs of hormone excess were present. Functional activity was determined as Cushing syndrome, virilization, or both. Age-dependent symptoms of virilization included premature pubarche, acne, voice changes, and hirsutism, and were subsequently referred to as virilization.

Tumor volume, tumor weight, and tumor maximum diameter were extracted from the histo-pathologic reports in the case of tumor resection (without neoadjuvant chemotherapy) or estimated from radiological images in the case of inoperability. Lymph node involvement and the Ki-67 index were defined histopathologically. For the Ki-67, the number of positive cells was counted for 100 cells per high-power field, and a percentage cut-off of >15% was used according to the five-item microscopic score [17,21].Distant metastases were detected by computed tomography and/or magnetic resonance imaging.

Complete remission (CR) was defined as the absence of any structural or functional evidence of disease. The COG staging system was used for the post-surgical tumor status [8,21,24]. Tumors were retrospectively stratified to ‘favorable’ (≤2 features) or ‘unfavorable’ (≥3 features) histology, according to the five-item microscopic score described by Picard et al. [17] and recommended by the EXPeRT group [21]. 

Data were included from 1997 until the 31st of December 2021.

### 2.2. Statistical Analyses

Kaplan–Meier estimates were used to calculate the overall survival (OS) and event-free survival (EFS), calculated from diagnosis to death of the disease without regard to other causes (OS) and to the event (EFS), defined as progression, relapse, or death of disease. Times for living patients were censored at the last follow-up.

A log-rank test was used to compare the groups. Categorical data were compared using a chi-squared test. The mean difference between subgroups was compared using a t-test, as appropriate, and in all other cases, the Mann–Whitney U was used. 

Univariate and multivariate analyses were done utilizing the Cox proportional hazards model with the OS and EFS as the outcome of interest. The multivariate model included prognostic factors analyzed in the univariate analysis. *P*-values ≤ 0.05 were considered evidence of a statistically significant association.

All analyses were performed using SPSS software, version 26.

## 3. Results

**Figure 1:**
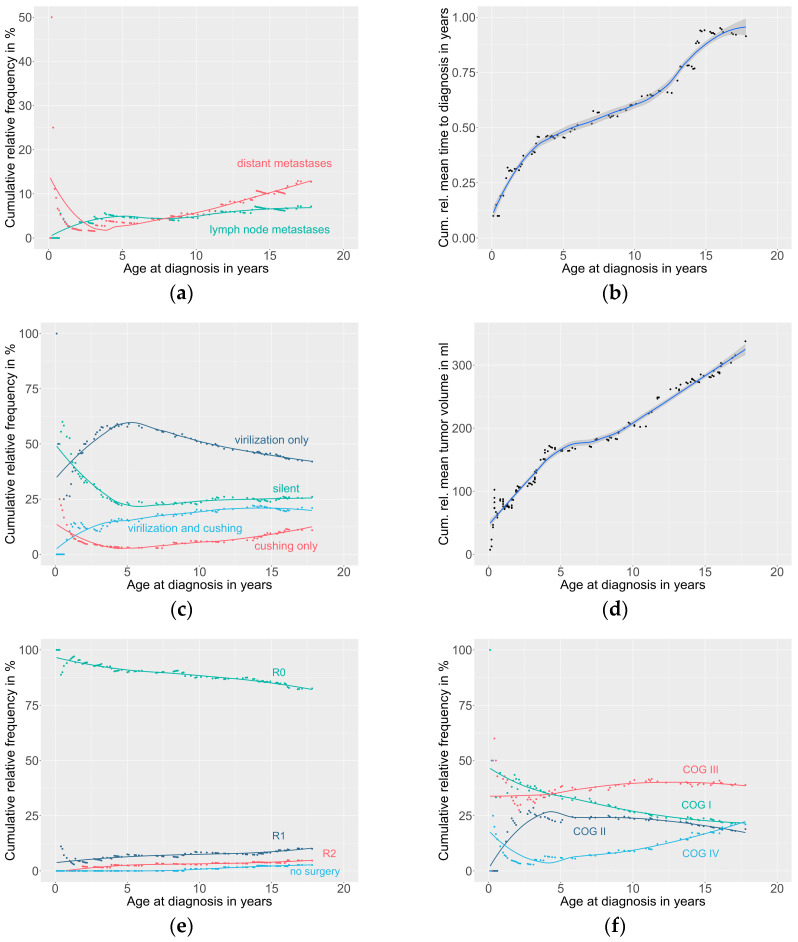
Details on patient characteristics by age: (**a**) Presence of metastases, (**b**) time to diagnosis, (**c**) endocrine phenotype, (**d**) tumor volume, (**e**) tumor resection status, and (**f**) COG stage for ACC/ACx.

The MET database registered 161 patients with ACTs [ACA *n* = 51 (31.7%), ACx *n* = 19 (11.8%), and ACC *n* = 91 (56.6%)] with a median age at diagnosis of 4.3 years (a range of 0.1–17.8) and a female to male ratio of 2.4:1. Retroperitoneal lymph node involvement and distant metastases were present in 11 (of 103; 10.7%) and 20 (of 106; 18.9%) of patients with ACCs/ACx. The patients aged ≥ 4 years more frequently presented with distant metastases compared to patients < 4 years (30.9% vs. 5.9%, *p* = 0.002). No difference was observed for retroperitoneal lymph node involvement in these age groups (8.2% vs. 13.0%, *p* = 0.640). The patient characteristics are detailed in Table 1, and the patient characteristics by age are in Figure 1.

The median time between the first symptoms and diagnosis was 8.8 months in the females and 16.3 months in the males (*p* = 0.155). The time to diagnosis was shorter in patients < 4 years compared to patients ≥ 4 years (5.5 months vs. 15.5 months, *p* = 0.002). The time to diagnosis increased linearly with age (0.5 months per year of age at diagnosis, R^2^ = 0.945, and *p* < 0.001). Virilization was more frequently present in patients < 4 years compared to patients ≥ 4 years (72.0% vs. 60.3%, *p* = 0.173), whereas Cushing syndrome was more frequent in patients ≥ 4 years (21.2% vs. 37.0%, *p* = 0.013). The details of the endocrine phenotype by age are given in Figure 1.

### 3.1. Neoadjuvant Treatment

In 20 (of 161; 12.4%) patients, one to six cycles of neoadjuvant chemotherapy [NN-1/NN-2, *n* = 15; cisplatin/etoposide/doxorubicin, *n* = 2; SIOP nephroblastoma, *n* = 2 (initially mistaken as nephroblastoma); SIOPEL hepatoblastoma, *n* = 1 (mistaken as hepatoblastoma)], with or without mitotane, were administered. In 16 (of 20; 80.0%) patients, tumor resection was performed after a median of four cycles of chemotherapy. The response evaluation after two to four cycles of chemotherapy demonstrated a partial response in 7 (of 19; 36.8%), stable disease in 4 (21.1%), and progressive disease in 8 (42.1%). In 4 (of 20; 20.0%) patients, neoadjuvant chemotherapy failed to achieve operability, and they received chemotherapy only. 

### 3.2. Surgical Management, Histopathological Evaluation, and Post-Surgical Staging

In 21 (of 155; 13.0%) patients, a biopsy was performed prior to resection. Biopsies were just as frequent in patients ≥ 4 years compared to patients < 4 years (16.3% vs. 10.7%, *p* = 0.435). 

A microscopically complete (R_0_) tumor resection was achieved in 124 (of 146; 84.9%) patients, whereas microscopic (R_1_) and macroscopic (R_2_) residues were left in 15 (10.3%) and 7 (4.8%) patients, respectively. The details of the histopathological evaluation are given in Table 2. R_0_ resection was achieved less frequently in patients ≥ 4 years compared to patients < 4 years (74.4% vs. 91.7%, *p* = 0.001). Intraoperative tumor spillage occurred in 21 (of 146; 14.4%) patients. Spillage was more frequent in patients ≥ 4 years (20.0% vs. 8.5%, *p* = 0.080). 

The median tumor volume was 160.8 cm^3^ (with a range of 0.5–3645.0), with significantly larger tumors in children ≥ 4 than <4 years of age (*p* < 0.001). The tumor volume increased linearly by 14.5 mL per year of age at diagnosis (R^2^ = 0.962, *p* < 0.001). On the histopathology, the Ki-67 index was >15% in 52 (of 133; 39.1%) patients, without differences between patients aged < 4 years and ≥4 years (35.8% vs. 42.4%, *p* = 0.547). The Wieneke index classified 56 (of 88; 63.6%) tumors as benign and 23 (26.1%) as malignant, while 9 (10.2%) tumors remained undetermined. The malignant histology was more frequent in patients aged ≥ 4 years compared to those <4 years (65.1% vs. 47.4%, *p* = 0.036). According to the five-item microscopic score, 62 (of 112; 55.4%) tumors were allocated to favorable and 50 (44.6%) to unfavorable histology. Unfavorable histology was just as frequent in patients ≥ 4 years compared to patients < 4 years (48.2% vs. 41.1%, *p* = 0.569). 

The post-surgical COG staging system in patients with ACCs and ACx allocated 20 (of 95; 21.1%) patients to stage I, 18 (18.9%) to stage II, 37 (38.9%) to stage III, and 20 (21.1%) to stage IV. Stage IV patients were older when compared to those with stages I, II, and III (*p* < 0.001).

### 3.3. Adjuvant Treatment in Patients with ACCs and ACx

In stage III patients with ACCs/ACx, the combination of chemotherapy and mitotane was given to seventeen (of thirty-five; 48.6%) patients and chemotherapy alone to two (5.7%). No adjuvant treatment was administered in 16 (45.7%) patients. In metastatic disease (stage IV), adjuvant treatment encompassed chemotherapy and mitotane in 9 (of 15; 60.0%) patients, chemotherapy alone in 2 (13.3%), and mitotane alone in 3 (20.0%). No adjuvant treatment was given to one (6.7%) stage IV patient. In 3 stage II cases with T4 tumors, mitotane was given postoperatively. Mitotane was administered over a median duration of 10.8 months (with a range of 0.5–21.6) in stage III and 4.2 months (with a range of 0.2–30) in stage IV cases. Adjuvant therapy was terminated due to side effects in 5 (of 34; 14.7%) patients.

The response evaluation after two to four cycles of adjuvant chemotherapy in 20 patients with residual tumor lesions demonstrated CR in one (5.0%), a partial response in 8 (40.0%), stable disease in 2 (10.0%), and progressive disease in 9 (45.5%). In 19 patients, adjuvant chemotherapy was given without a residual tumor.

### 3.4. Outcome and Subsequent Events in ACCs and ACx

**Figure 2:**
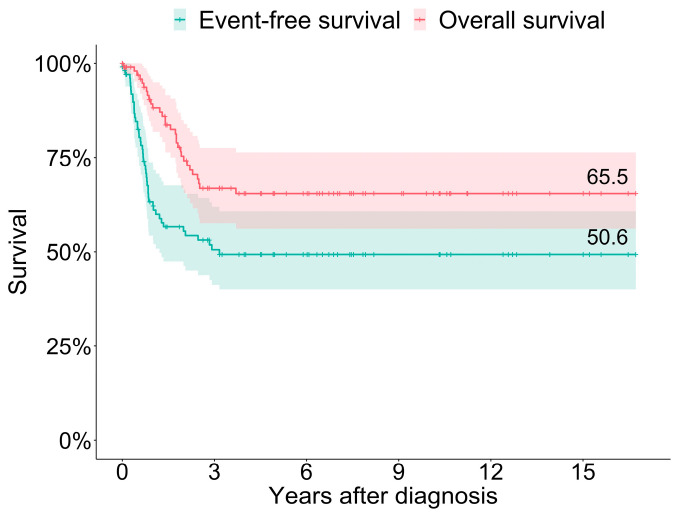
Probabilities of overall and event-free survival in 110 children and adolescents with ACCs and ACx.

The median duration of the follow-up was 2.6 years (with a range of 0–16.7) and a mean duration of 4.5 years. The three-year OS and EFS estimates were 65.5% and 50.6% (Figure 2). 

Thirty (of 110; 27.3%) patients died due to the ACC/ACx after a median time of 17.2 months (with a range of 0.5–44.4) after diagnosis, including those 4 patients with inoperable tumors following neoadjuvant chemotherapy. Patients succumbing to the disease were significantly older than the surviving patients (median 11.1 vs. 3.2, *p* < 0.001). Progression or relapse occurred at a median of 7.2 months (with a range of 0–15.4) and 8.2 months (with a range of 1.0–37.9) after diagnosis in 47 (42.7%) patients. 

The events were locoregional recurrences in 8 (of 110; 7.3%) patients, distant metastases in 18 (16.4%), combined relapses in 6 (5.5%), and progression in 15 (13.6%). The events following complete tumor resection (R_0_) were locoregional recurrences in 8 (of 110; 7.3%) patients, distant metastases in 18 (16.4%), and combined relapses in 6 (5.5%).

Of 34 patients with R_1/2_ tumor resection and/or tumor spillage, 22 (64.7%) patients suffered from a subsequent event, which was a locoregional relapse in 6 (of 22; 27.3%) patients, a distant relapse in 3 (13.6%), a combined relapse in 2 (9.1%), and progression in 11 (50.0%). 

### 3.5. Outcome for the Clinical Factors and Assessment of Risk Factors in Patients with ACCs and ACx

**Figure 3:**
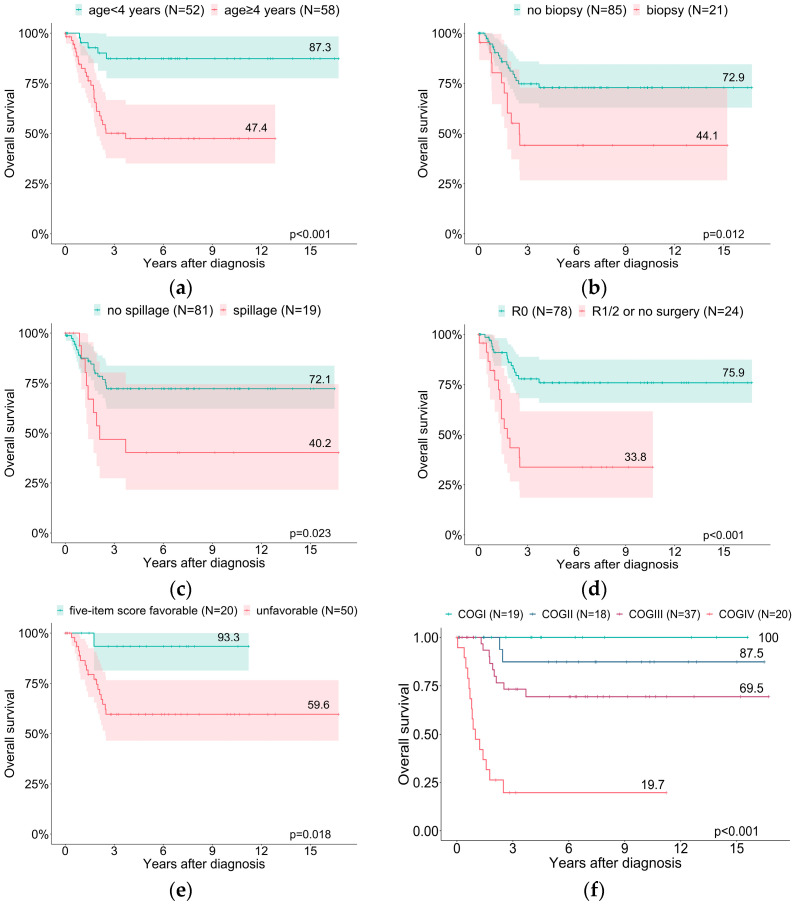
Probabilities of overall survival relating to age (**a**), initial biopsy (**b**), tumor spillage (**c**), tumor resection status (**d**), five-item microscopic score (**e**), and COG stage (**f**).

In the univariate analysis, age at diagnosis ≥ 4 years, an unfavorable five-item microscopic score, tumor spillage, incomplete tumor resection, a tumor volume of ≥ 200 mL, a Ki67 index of >15%, and COG stages III/IV significantly impacted the negative prognostic factors upon the OS and EFS (Figure 3, Table 3). The initial biopsy represented a significant determinant of the OS (HR 2.5, a 95% CI, 1.2–5.3, and *p* = 0.016) but not of the EFS (HR 1.8, a 95% CI, 1.0–3.5, and *p* = 0.068).

**Figure 4:**
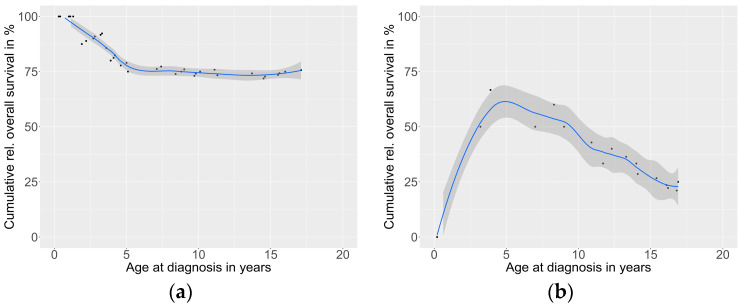
Overall survival in COG stage III (**a**) and stage IV (**b**) patients by age.

The Cox regression determined that the risk of death was 8-fold higher for a tumor volume of ≥200 mL compared to that of <200 mL (*p* < 0.001). The overall survival of patients with COG stage III decreased linearly by 1.5% per year of age at diagnosis (R^2^ = 0.662, *p* < 0.001) and in stage IV patients by 2.9% (starting at 3.2 years of age; R^2^ = 0.850, *p* < 0.001) (Figure 4).

The age at diagnosis, five-item score, and COG III/IV stages were considered in a multivariate Cox regression model. An unfavorable five-item score and COG stage IV significantly impacted the prognostic factors upon the EFS and OS in the final model (Table 4).

## 4. Discussion

Herein, we have reported the outcome of children and adolescents with ACTs registered in the MET studies. Our data confirm that ACCs/ACx in children and adolescents are associated with unfavorable outcomes (3-year OS: 65.5%; EFS: 50.6%) [6,10,18]. An age ≥ 4 years, the initial biopsy, tumor spillage, incomplete resection, unfavorable histology, and COG stages III and IV impacted the negative prognostic factors upon the OS and EFS. 

Age defines the clinical presentation and prognosis in children and adolescents with ACTs [17,19,20,25,26]. Patients aged ≥ 4 years more frequently presented with advanced tumor stages, including distant metastases, compared to younger patients. Noteworthy is that complete tumor resection was more frequently achieved in younger patients, while tumor spillage occurred with increasing frequency in older patients. 

Tumor spillage has previously been associated with a worse prognosis and almost fatal outcomes [19,27]. In line with this, tumor spillage negatively impacted the overall and event-free survival in our study. The impact of an initial biopsy is still under debate. In adults, an initial biopsy does not affect survival [28], while poorer outcomes following a biopsy were reported in children [18,23]. The EXPeRT/PARTNER guideline strongly recommends avoiding an initial tumor biopsy whenever possible. This is corroborated by our data; an initial biopsy significantly impacted the overall survival.

Different series have shown that complete tumor resection is a major prognostic factor and may cure the patient [6,13,29]. Incomplete resection was also associated with worse outcomes in our study (an OS of 33.8%). The resection status has been incorporated into the modified postsurgical COG staging system [8]. It relies on the tumor size, status of resection, and status of metastases. It, thereby, encompasses most clinical prognostic factors in children and adolescents with ACTs [10,13,25,26]. Accordingly, in our study, the COG stage correlated with the outcome (OS COG I 100%, COG II 87.5%, COG III 69.5%, and COG IV 19.7%), confirming previous studies [18,20]. However, in the ARAR0332 study, the overall survival of stage II patients with completely resected large tumors was only 78.8%. 

This nicely illustrates the extraordinary challenge in children and adolescents with ACTs: the pathological stratification of pediatric ACTs is difficult, which hinders therapeutic stratification. In the ARAR0332 study, the pathology review was based on the Wieneke index stratification [12]. However, the Wieneke index considers a number of criteria and is observer-dependent [21]. Picard and colleagues described a five-item microscopic score classifying ACTs in ‘favorable’ and ‘unfavorable’ histology [17]. The EXPeRT/PARTNER guideline recommends exploring the five-item score in pediatric ACTs [21]. This is linked to the hope of improving the stratification of localized pediatric ACTs: on the one hand, to limit the use of systemic treatment in patients with favorable tumor histology and, on the other hand, to explore systemic treatment regimens in patients with unfavorable histology, and indeed, unfavorable histology by means of the five-item microscopic score was associated with inferior overall and event-free survival in our cohort. It should not go unmentioned that the score was calculated retrospectively in our study. Based on a systematic review of the literature, Riedmeier and colleagues recently assessed a modified S-GRAS score for pediatric ACTs [25]. The final score encompassed the tumor stage, grade (Ki67 index/rate of mitosis), resection status, age, and hormone-related symptoms. The pediatric S-GRAS score stratifies pediatric ACT patients into four groups. However, the correlation of these groups with clinical outcomes needs to be confirmed in future studies. 

In the EXPeRT/PARTNER guideline, therapeutic recommendations allow for a resection status and COG stage and, thus, distinguish between resected and advanced-stage ACTs [21]. Starting with stage II patients and non-benign histology, mitotane therapy should be considered. In patients with R_1/2_ resection and/or spillage, as well as in stage III and IV patients, chemotherapy plus mitotane is recommended. In contrast, the MET studies advised systemic therapy only for metastatic patients (locoregional lymph nodes and/or distant metastases). The MET studies were not designed and powered to prove the efficacy of chemotherapy plus mitotane. In particular, the type and length of therapy were virtually decided on an individual basis by treating physicians, as the GPOH-MET 97 study was not an interventional study according to modern standards. We previously reported on children and adolescents with ACCs and mitotane therapy [30]. Especially in patients with advanced disease, the treatment was not administered as advised, due to inefficacy and/or toxicity. Our analyses showed a survival benefit for patients receiving mitotane therapy (OS HR 3.6, a 95% CI 1.6–8.1, and *p* = 0.002; EFS HR 1.9, a 95% CI 1.0–3.5, and *p* = 0.037). These data need to be interpreted carefully; only patients in good clinical condition received chemotherapy plus mitotane. In the ARAR0332 protocol, with the combination of chemotherapy plus mitotane, the 5-year overall survival in stage III patients was excellent (94.7%) but poor (7.1%) in stage IV patients receiving the same regimen [20]. This clearly demonstrates the important limitations of systemic therapy in terms of efficacy and toxicity. 

To better define the biology of the disease is of crucial importance for the best prognostic stratification and ultimately for improving the outcome in children and adolescents with ACTs. Thus, progress in our understanding of pathogenesis, as well as additional therapeutic avenues, are needed. Recent findings suggest that somatic mutations and methylation markers may predict prognosis [20,26,31]. Comprehensive genetic and molecular analyses were not yet included in the MET studies.

In summary, international prospective studies are needed to explore important, open questions in children and adolescents with ACTs, such as the role of adjuvant/neo-adjuvant chemotherapy, the best chemotherapy regimen, the best indications, and treatment of poor-responding, metastatic, and relapsed ACT patients. 

## 5. Conclusions

Age defines the clinical presentation and prognosis in children and adolescents with ACTs. The outcome in patients with advanced and metastatic ACTs was poor and was best predicted by the COG stage and five-item microscopic score. International efforts are needed to improve our understanding of the pathogenesis, introduce new therapeutic avenues, and ultimately increase survival in children and adolescents with ACTs.

## Figures and Tables

**Table 1:** Patient characteristics of 161 children and adolescents with adrenocortical tumors.

Characteristics	<4 Years		≥4 Years		Total		*p*-Value
	*n*	%	*n*	%	*n*	%	
Diagnoses							
ACC	37	47.4	54	65.1	91	56.5	0.009
ACx	15	19.2	4	4.8	19	11.8	
ACA	26	33.3	25	30.1	51	31.7	
Sex							
Female	48	61.5	65	78.3	113	70.2	0.031
Male	30	38.5	18	21.7	48	29.8	
Age (years) at diagnosis							
Median	1.4		11.3		4.3		
Range	0.1–3.9		4.1–17.8		0.1–17.8		
Distant metastases in							
ACC/ACx	3	5.8	17	29.3%	20	18.2	0.003
Lung	3		15		18		
Liver	0		5		5		
Bone	0		3		3		
Central nervous system	0		1		1		
No	49		41		90		
Not reported	0		0		0		
Symptomatic interval (months) prior to diagnosis							
Median	3.6		6.0			4.8	0.002
Range	0–30.0		0–169.2			0–169.2	
Endocrine phenotype							
Cushing syndrome	2	3.4	11	18.0	13	10.9	0.004
Virilization *	33	56.9	17	27.9	50	42.0	
Combined	9	15.5	16	26.2	25	21.0	
Silent	14	24.1	17	27.9	31	26.1	
Not reported	20		22		42		

* Symptoms of virilization were age-dependent, including premature pubarche, acne, hirsutism, and voice changes.

**Table 2:** Histopathological features and COG stage in 110 children and adolescents with an ACC and ACx.

	<4 Years		≥4 Years		Total		*p*-Value
	*n*	%	*n*	%	*n*	%	
Tumor volume (cm^3^)							
Median	113.0		336.5		160.8		<0.001
Range	0.5–1260.0		11.4–3645.0		0.5–3645.0		
Tumor weight (g)							
Median	123.5		303.5		215.0		0.008
Range	9.0–1214.0		11.7–3300.0		9.0–3300.0		
Tumor diameter (cm)							
maximum							
Median	7.4		10.5		8.5		
Range	1.0–19.5		4.0–30.0		1.0–30.0		<0.001
Resection status							
Complete	43	89.6	35	64.8	78	76.5	0.023
Microscopic residue	3	6.3	10	18.5	13	12.7	
Macroscopic residue	2	4.2	5	9.3	7	6.9	
No surgery	0		4	7.4	4	3.9	
Vena cava invasion							
Yes	9	18.0	16	29.1	25	23.8	
No	41	82.0	39	70.9	80	76.2	0.270
Venous invasion							
Yes	26		30		56	70.9	
No	13	66.7	10	75.0	23	29.1	0.570
Not reported	13	33.3	18	25.0	31		
Tumor necrosis							
Yes	34		40		74		
No	12	73.9	7	85.1	19	79.6	0.280
Not reported	6	26.1	11	14.9	17	20.4	
Ki-67 index							
≤15%	22	48.9	17	37.8	39	43.3	0.395
>15%	23	51.1	28	62.2	51	56.7	
Wieneke score							
<3	11	45.8	7	26.9	18	36.0	
=3	6	25.0	3	11.5	9	18.0	0.069
>3	7	29.2	16	61.5	23	46.0	
Five-item score							
≤2 features	11	32.4	9	25.0	20	28.6	
>2 features	23	67.6	27	75.0	50	71.4	0.677
COG stage for ACC/ACx							
Stage I	16	35.6	4	8.0	20	21.1	
Stage II	11	24.4	7	14.0	18	18.9	
Stage III	15	33.3	22	44.0	37	38.9	
Stage IV	3	6.7	17	34.0	20	21.1	<0.001

**Table 3:** Univariate analysis for risk factors for unfavorable overall survival (OS) and event-free survival (EFS) for 110 children and adolescents with ACCs and ACx.

		OS			EFS	
Univariate Analysis	HR	95% CI	*p*	HR	95% CI	*p*
Age at diagnosis ≥ 4 years	5.4	2.1–14.1	0.001	3.0	1.6–5.9	0.001
Virilization	0.6	0.3–1.3	0.205	0.8	0.4–1.6	0.562
Cushing syndrome	1.6	0.8–3.4	0.217	1.5	0.8–2.8	0.165
Five-item score, unfavorable	7.5	1.0–58.2	0.047	4.6	1.4–15.2	0.012
Initial biopsy	2.5	1.2–5.3	0.016	1.8	1.0–3.5	0.068
Tumor spillage	2.4	1.1–5.4	0.028	2.2	1.1–4.4	0.019
R_1/2_ resection or no surgery	4.0	1.9–8.4	<0.001	2.7	1.5–5.0	0.001
Tumor volume ≥ 200 mL	8.0	2.8–23.0	<0.001	3.7	1.9–7.1	<0.001
Ki67 index > 15%	2.9	1.1–8.0	0.034	3.3	1.5–7.2	0.003
COG stage III or IV	10.3	2.4–43.7	0.002	6.4	2.5–16.4	<0.001
COG stage IV	10.6	4.7–23.7	<0.001	5.2	2.7–9.9	<0.001

**Table 4:** Cox regression analyses for overall and event-free survival in children and adolescents with ACCs and ACx.

	OS			EFS		
Multivariable Analysis	HR	95% CI	*p*	HR	95% CI	*p*
Age at diagnosis ≥ 4 years	2.8	0.8–10.3	0.127	1.7	0.7–4.2	0.285
Five-item score > 2 features	7.5	1.0–58.2	0.047	5.8	1.4–24.8	0.018
COG stage III	2.7	0.5–14.3	0.231	3.2	1.0–10.9	0.057
COG stage IV	10.5	2.1–52.8	0.004	7.6	2.2–26.0	0.001

## Data Availability

The data presented in this study are available on request from the corresponding author. The data are not publicly available due to restrictions.

## References

[1] Siegel D.A., King J., Tai E., Buchanan N., Ajani U.A., Li J. (2014). Cancer Incidence Rates and Trends Among Children and Adolescents in the United States, 2001–2009. Pediatrics.

[2] Dall’Igna P., Virgone C., De Salvo G.L., Bertorelle R., Indolfi P., De Paoli A., Buffa P., Conte M., Esposito G., Inserra A. (2014). Adrenocortical tumors in Italian children: Analysis of clinical characteristics and P53 status. Data from the national registries. J. Pediatr. Surg..

[3] Wasserman J.D., Novokmet A., Eichler-Jonsson C., Ribeiro R.C., Rodriguez-Galindo C., Zambetti G.P., Malkin D. (2015). Prevalence and Functional Consequence of *TP53* Mutations in Pediatric Adrenocortical Carcinoma: A Children’s Oncology Group Study. J. Clin. Oncol..

[4] Ribeiro R.C., Sandrini F., Figueiredo B., Zambetti G.P., Michalkiewicz E., Lafferty A.R., DeLacerda L., Rabin M., Cadwell C., Sampaio G. (2001). An inherited p53 mutation that contributes in a tissue-specific manner to pediatric adrenal cortical carcinoma. Proc. Natl. Acad. Sci. USA.

[5] Mastellaro M.J., Seidinger A.L., Kang G., Abrahão R., Miranda E.C.M., Pounds S.B., Cardinalli I.A., Aguiar S.S., Figueiredo B.C., Rodriguez-Galindo C. (2017). Contribution of the *TP53* R337H mutation to the cancer burden in southern Brazil: Insights from the study of 55 families of children with adrenocortical tumors. Cancer.

[6] Michalkiewicz E., Sandrini R., Figueiredo B., Miranda E.C., Caran E., Oliveira-Filho A.G., Marques R., Pianovski M.A., Lacerda L., Cristofani L.M. (2004). Clinical and Outcome Characteristics of Children With Adrenocortical Tumors: A Report From the International Pediatric Adrenocortical Tumor Registry. J. Clin. Oncol..

[7] Wagner J., Portwine C., Rabin K., Leclerc J.-M., Narod S.A., Malkin D. (1994). High Frequency of Germline p53 Mutations in Childhood Adrenocortical Cancer. JNCI J. Natl. Cancer Inst..

[8] Ribeiro R.C., Pinto E.M., Zambetti G.P., Rodriguez-Galindo C. (2012). The International Pediatric Adrenocortical Tumor Registry initiative: Contributions to clinical, biological, and treatment advances in pediatric adrenocortical tumors. Mol. Cell Endocrinol..

[9] Rodriguez-Galindo C., Figueiredo B.C., Zambetti G.P., Ribeiro R.C. (2005). Biology, clinical characteristics, and management of adrenocortical tumors in children. Pediatr. Blood Cancer.

[10] Cecchetto G., Ganarin A., Bien E., Vorwerk P., Bisogno G., Godzinski J., Dall’Igna P., Reguerre Y., Schneider D., Brugières L. (2016). Outcome and prognostic factors in high-risk childhood adrenocortical carcinomas: A report from the European Cooperative Study Group on Pediatric Rare Tumors (EXPeRT). Pediatr. Blood Cancer.

[11] Dehner L.P. (2003). Pediatric Adrenocortical Neoplasms. Am. J. Surg. Pathol..

[12] Wieneke J.A., Thompson L.D.R., Heffess C.S. (2003). Adrenal Cortical Neoplasms in the Pediatric Population. Am. J. Surg. Pathol..

[13] Zambaiti E., Duci M., De Corti F., Gamba P., Dall’Igna P., Ghidini F., Virgone C. (2020). Clinical prognostic factors in pediatric adrenocortical tumors: A meta-analysis. Pediatr. Blood Cancer.

[14] Chatterjee G., DasGupta S., Mukherjee G., Sengupta M., Roy P., Arun I., Datta C., Mishra P.K., Banerjee S., Chatterjee U. (2015). Usefulness of Wieneke criteria in assessing morphologic characteristics of adrenocortical tumors in children. Pediatr. Surg. Int..

[15] Jehangir S., Nanjundaiah P., Sigamani E., Burad D., Manipadam M.T., Lea V., Ly T., Holland A.J.A. (2018). Pathological prognostication of paediatric adrenocortical tumours: Is a gold standard emerging?. Pediatr. Blood Cancer.

[16] Teinturier C., Pauchard M.S., Brugieres L., Landais P., Chaussain J.L., Bougneres P.F. (1999). Clinical and prognostic aspects of adrenocortical neoplasms in childhood. Med. Pediatr. Oncol..

[17] Picard C., Orbach D., Carton M., Brugieres L., Renaudin K., Aubert S., Berrebi D., Galmiche L., Dujardin F., Leblond P. (2018). Revisiting the role of the pathological grading in pediatric adrenal cortical tumors: Results from a national cohort study with pathological review. Mod. Pathol..

[18] Picard C., Faure-Conter C., Leblond P., Brugières L., Thomas-Teinturier C., Hameury F., Defachelles A., Verschuur A., Brisse H.J., Sarnacki S. (2019). Exploring heterogeneity of adrenal cortical tumors in children: The French pediatric rare tumor group (Fracture) experience. Pediatr. Blood Cancer.

[19] McAteer J.P., Huaco J.A., Gow K.W. (2013). Predictors of survival in pediatric adrenocortical carcinoma: A Surveillance, Epidemiology, and End Results (SEER) program study. J. Pediatr. Surg..

[20] Rodriguez-Galindo C., Krailo M.D., Pinto E.M., Pashankar F., Weldon C.B., Huang L., Caran E.M., Hicks J., McCarville M.B., Malkin D. (2021). Treatment of Pediatric Adrenocortical Carcinoma With Surgery, Retroperitoneal Lymph Node Dissection, and Chemotherapy: The Children’s Oncology Group ARAR0332 Protocol. J. Clin. Oncol..

[21] Virgone C., Roganovic J., Vorwerk P., Redlich A., Schneider D.T., Janic D., Bien E., López-Almaraz R., Godzinski J., Osterlundh G. (2021). Adrenocortical tumours in children and adolescents: The EXPeRT/PARTNER diagnostic and therapeutic recommendations. Pediatr. Blood Cancer.

[22] Hubertus J., Boxberger N., Redlich A., von Schweinitz D., Vorwerk P. (2012). Surgical Aspects in the Treatment of Adrenocortical Carcinomas in Children: Data of the GPOH-MET 97 Trial. Klin. Pädiatrie.

[23] Redlich A., Boxberger N., Strugala D., Frühwald M.C., Leuschner I., Kropf S., Bucsky P., Vorwerk P. (2012). Systemic Treatment of Adrenocortical Carcinoma in Children: Data from the German GPOH-MET 97 Trial. Klin. Pädiatrie.

[24] Sandrini R., Ribeiro R.C., DeLacerda L. (1997). Childhood Adrenocortical Tumors^1^. J. Clin. Endocrinol. Metab..

[25] Riedmeier M., Decarolis B., Haubitz I., Reibetanz J., Wiegering A., Härtel C., Schlegel P.-G., Fassnacht M., Wiegering V. (2022). Assessment of prognostic factors in pediatric adrenocortical tumors: A systematic review and evaluation of a modified S-GRAS score. Eur. J. Endocrinol..

[26] Pinto E.M., Rodriguez-Galindo C., Pounds S.B., Wang L., Clay M.R., Neale G., Garfinkle E.A., Lam C.G., Levy C.F., Pappo A.S. (2017). Identification of Clinical and Biologic Correlates Associated With Outcome in Children With Adrenocortical Tumors Without Germline *TP53* Mutations: A St Jude Adrenocortical Tumor Registry and Children’s Oncology Group Study. J. Clin. Oncol..

[27] Gulack B.C., Rialon K.L., Englum B.R., Kim J., Talbot L.J., Adibe O.O., Rice H.E., Tracy E.T. (2015). Factors associated with survival in pediatric adrenocortical carcinoma: An analysis of the National Cancer Data Base (NCDB). J. Pediatr. Surg..

[28] Williams A.R., Hammer G.D., Else T. (2014). Transcutaneous biopsy of adrenocortical carcinoma is rarely helpful in diagnosis, potentially harmful, but does not affect patient outcome. Eur. J. Endocrinol..

[29] Riedmeier M., Decarolis B., Haubitz I., Müller S., Uttinger K., Börner K., Reibetanz J., Wiegering A., Härtel C., Schlegel P.-G. (2021). Adrenocortical Carcinoma in Childhood: A Systematic Review. Cancers.

[30] Kuhlen M., Mier P., Kunstreich M., Lessel L., Schneider D., Brecht I., Schewe D.M., Frühwald M.C., Vorwerk P., Redlich A. (2022). Key factors for effective mitotane therapy in children with adrenocortical carcinoma. Endocr.-Relat. Cancer.

[31] Pinto E.M., Chen X., Easton J., Finkelstein D., Liu Z., Pounds S., Rodriguez-Galindo C., Lund T.C., Mardis E.R., Wilson R.K. (2015). Genomic landscape of paediatric adrenocortical tumours. Nat. Commun..

